# Definition and characteristics of climate-adaptive cities: a systematic review

**DOI:** 10.1186/s12889-024-18591-x

**Published:** 2024-04-30

**Authors:** Arezoo Yari, Alireza Mashallahi, Hamidreza Aghababaeian, Mohsen Nouri, Nidhi Yadav, Arefeh Mousavi, Shiva Salehi, Abbas Ostadtaghizadeh

**Affiliations:** 1 Social Determinants of Health Research Center, Research Institute for Health Development, Kurdistan University of Medica Sciences, Sanandaj, Iran; 2 Present Address: Department of Health in Emergencies and Disasters, School of Public Health, Tehran University of Medical Sciences, Poorsina Ave, Tehran, 14177-43578 I.R Iran; 3 Center for Climate Change and Health Research (CCCHR), Dezful University of Medical Sciences, Dezful, Iran; 4 International Institute of Health Management Research, Delhi, India; 5 Social Determinants of Health Research Center, Isfahan University of Medical Sciences, Isfahan, Iran; 6 Department of Nursing, Faculty of Nursing and Midwifery, Tehran Medical Sciences, Islamic Azad University, Tehran, Iran; 7 Department of Health in Emergencies and Disasters, School of Medicine, Kurdistan University of Medical Sciences, Sanandaj, Iran; 8 Spiritual Health Research Center, Iran University of Medical Sciences, Tehran, Iran; 9 Climate Change and Health Research Center (CCHRC), Institute for Environmental Research (IER), Tehran University of Medical Sciences, Tehran, Iran

**Keywords:** Climate change, Climate-adaptive cities, Adaptation, Resilience, Resource management, Low-carbon economy

## Abstract

**Background:**

Cities, as frontline responders to climate change, necessitate a precise understanding of climate-adaptive features. This systematic review aims to define and outline the characteristics of climate-adaptive cities, contributing vital insights for resilient urban planning.

**Methods:**

This systematic review, initiated on March 6, 2018, and concluded on August 26, 2021, involved reviewing multiple electronic databases based on the study's objectives. The Critical Appraisal Skills Program (CASP) tool was used for quality assessment and critical evaluation of articles retrieved through a comprehensive and systematic text search. Descriptive and thematic analyses were conducted to extract definitions, features, and characteristics of climate-adaptive cities.

**Results:**

Out of 6104 identified articles, 38 articles met the inclusion criteria. In total, 20 definitions and 55 features for climate-adaptive cities were identified in this review. Codes were categorized into two categories and ten subcategories. The categories included definitions and features or characteristics of climate-adaptive cities.

**Conclusion:**

A climate-adaptive city, as derived from the findings of this study, is a city that, through effective resource management, future-oriented planning, education, knowledge utilization, innovation in governance and industry, decentralized management, and low-carbon economy, leads to the adaptability, resilience, sustainability, and flexibility of the capacity of individuals, communities, institutions, businesses, and systems within a city against all climate change impacts and reduces their negative consequences.

## Background

Climate change imposes greater stress on urban areas [1]. Urban areas, encompassing metropolitan and suburban regions, accommodate the majority of the global population [1, 2] and are accountable responsible for approximately 40% of greenhouse gas emissions [1] and over 70% of global CO_2_ emissions [2]. These statistics are projected to rise in the future [1]. In addition to greenhouse gas emissions, urban areas face challenges such as excessive energy and resource consumption, waste generation, crime, social and cultural instability, and the ongoing global population growth, all contributing to the complexities of climate change impacts [3]. Consequently, cities are at the forefront of addressing climate change challenges [1, 4], with many researchers considering them indispensable in this regard [5].

Modern human activities exacerbate various climate phenomena, including global warming, UHI (Urban Heat Island) effect, heatwaves, and droughts [6]. Climate change and its repercussions are among humanity's major concerns, posing significant challenges to global sustainable development [7]. The rapid pace of climate change, coupled with its pervasive and detrimental effects on the environment, economy, and public health, underscores the critical importance of addressing climate-related issues [8].

The recent escalation in temperatures, driven by global warming and intensified heat in urban centers due to the UHI effect, profoundly impacts urban life, especially during warmer seasons [6]. These climatic shifts not only pose significant health risks but also endanger vital necessities such as clean air, water, food, and shelter [9]. Moreover, climate change exacerbates poverty and marginalization, particularly among vulnerable populations [10]. The increasing global population further exacerbates climate-related challenges, including the UHI effect and urban heat issues [6]. The consensus on global warming's human-induced nature and the persistence of trends in energy consumption, development, and population growth emphasize the urgency of addressing climate change [10].

Climate change, urbanization, and aging populations in many regions are expected to heighten the risk of heat-related illnesses, particularly due to heat exposure [11]. Furthermore, climate change leads to more frequent and severe weather events, such as droughts, storms, precipitation, and heatwaves, contributing to social, economic, and environmental disruptions globally [12]. The anthropogenic nature of climate change underscores the need for adaptation efforts to mitigate its impacts [13]. Adaptation involves enhancing resilience and reducing vulnerability to observed or anticipated climate changes [14, 15]. Urban adaptation capacity, influenced by economic, social, and environmental factors, plays a crucial role in determining cities' ability to respond effectively to climate change [16].

In this context, adaptation capacity refers to the ability of a system to adjust to climate change, including climate variability and temperature thresholds, in order to mitigate potential damages, seize opportunities, and cope with resulting consequences [17]. Enhancing adaptation to climate change in urban areas involves implementing various methods. Adaptation capacity encompasses the ability of stakeholders to absorb and recover from the effects of climate change while also leveraging new opportunities to increase adaptability. Factors influencing the capacity for adaptation to climate change include the economic, social, and environmental characteristics of each region. These characteristics may have general applicability across regions or be specific to certain areas facing distinct levels of risk from climate change [18].

Given the adverse impacts of climate change, efforts to develop practical adaptation strategies have gained traction, shifting the focus from understanding vulnerabilities to implementing actionable plans [19]. The aim of global adaptation agreements is to bolster resilience, reduce vulnerability, and support sustainable development [20]. Community-based adaptation initiatives, integrated into urban policies at local and national levels, foster public participation and enhance urban resilience [21]. Notably, some communities have initiated measures to mitigate global warming impacts, such as assessing the role of vegetation and water surfaces in mitigating thermal effects [22].

Urban areas face significant challenges in creating climate adaptation conditions for their residents, necessitating effective urban climate change programs [23]. Social, economic, governmental, and environmental factors play pivotal roles in driving or hindering the development of such programs [24]. While large and affluent cities actively engage in climate planning, vulnerable cities and individuals with high exposure to climate impacts often have limited involvement [1].

The introduction underscores the imperative of understanding urban adaptation to climate change and aims to establish a foundational understanding of climate-adaptive cities. Despite various definitions of climate adaptation, those specifically addressing climate-adaptive cities remain limited and lack comprehensiveness. Through this study, we seek to identify the distinctive features and characteristics that define cities adept at responding to climate change challenges.

The implications of this research extend beyond academia, offering practical insights for urban policy and planning. By elucidating climate adaptation intricacies in urban areas, this study contributes to the development of robust mitigation and adaptation strategies, ultimately enhancing urban resilience and safeguarding residents from the adverse impacts of climate change. Additionally, the findings are poised to guide policymakers and urban planners in formulating more effective strategies, fostering sustainable and resilient urban ecosystems amid evolving climate conditions.

## Materials and methods

This study is part of a review that examines the concepts, characteristics, components, challenges, and implementation strategies of climate-adaptive cities. In this study, only the definition and characteristics of adaptive cities are presented. The following steps were taken for this study, which was then evaluated using the PRISMA checklist.

### Inclusion and exclusion criteria

This study encompassed published articles and books addressing the definition, characteristics, and features of climate-adaptive cities within the scope of the research questions. The inclusion of articles and documents was not restricted by time, covering works available until March 6, 2018. It is crucial to clarify that while there was no limitation on the publication date of included materials, the retrieval process and study focus encompassed documents available until the specified date. Following the collection of studies, the examination of entered studies commenced and persisted until August 26, 2021. Excluded were studies not addressing the definitions or characteristics of climate-adaptive cities, those solely examining other components, studies unrelated to events, disasters, accidents, or crises, and articles not related to a human population residing in a specific geography. Additionally, documents not available in full text and not relevant to our research topic, as well as studies not in English, were excluded.

### Databases and search strategy

This systematic review utilized available books, manuals, guidelines, and scientific resources, as well as electronic searches on various websites and databases available on the Internet, including PubMed, Web of Science, EMBASE, Cochrane Library, Scopus, ScienceDirect, and Google Scholar, without any limitations on the date or type of study. The language of the research in the above-mentioned databases was English. In addition to these databases, reputable international websites, such as those affiliated with the United Nations (UNDP, UN-HABITAT, UNISDR, UNEP), were also examined. Moreover, articles on Google Scholar were searched manually. Reading the articles' references and using the snowball mechanism were other methods used to find relevant articles. This study was conducted on March 6, 2018.

The following English keywords and their similar terms extracted from the MeSH database or the Tazaroos database, which is specifically designed to identify synonymous terms, were used. It should be noted that consultation and agreement with experts and stakeholders were carried out before the search regarding the keywords and types of terms. In general, only two groups of words were used to increase the study's sensitivity, which are:

- Group 1 keywords: City, Urban, Municipal, Civil, Burgh
- Group 2 keywords: Adapt*, Cop*, Resil*, Accommodat*

### Study selection

**Fig. 1:**
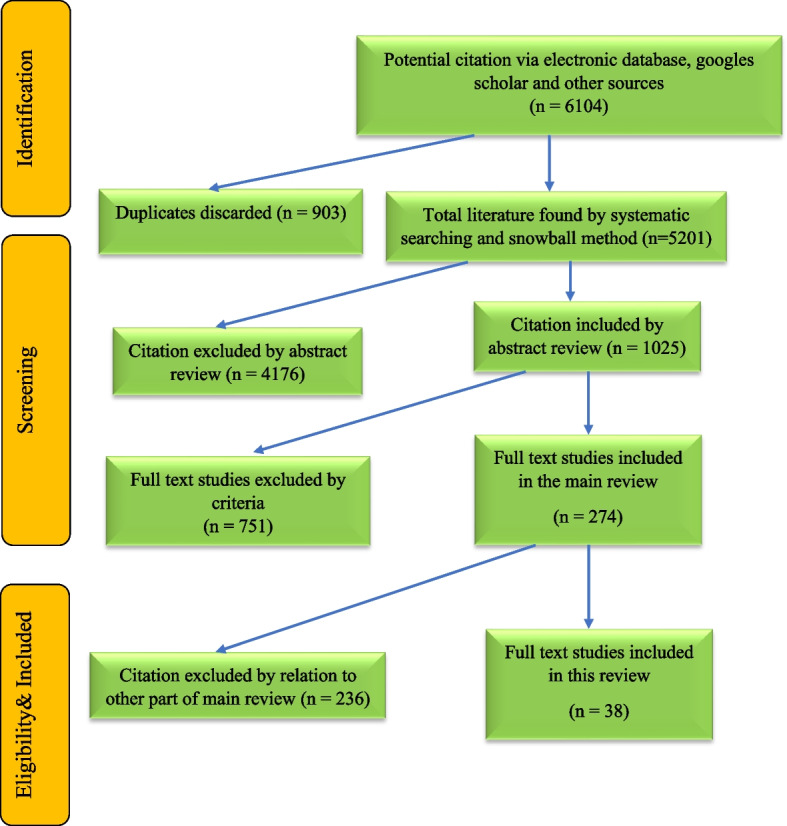
Flow chart diagram of the screening process for included studies on the definitions and characteristics of climate-adaptive cities

Based on the inclusion and exclusion criteria, the researchers (AY, AM, HA, MN, NY, AM, SHS) screened the titles and abstracts of the retrieved articles using the EndNote software to find relevant articles. Then, the full-text of the selected articles was independently reviewed by two researchers (AY, AM). In case of disagreement between the two researchers, a third researcher (AOT) resolved the differences and helped them make the best selection. The process of reviewing and selecting articles is shown in Fig. 1.

### Quality appraisal and data extraction

The quality of the articles was evaluated by the researchers using the Critical Appraisal Skills Program (CASP), which provides a systematic way to evaluate the overall quality, reliability, and quality of different study designs [25, 26]. CASP provides a structured framework for assessing key aspects such as research methodology, sampling, data analysis, and result reporting.

Each article underwent a detailed evaluation based on specific parameters outlined in the CASP criteria. These parameters encompassed methodological rigor, validity of findings, appropriateness of statistical methods, and the clarity of reporting. The evaluation process resulted in the categorization of articles into three quality groups: low, moderate, and high. Documents and articles that were categorized in the low-quality group were excluded, and articles categorized in the moderate and high-quality groups were included in the study. This thorough quality appraisal process aimed to uphold the integrity and credibility of the included studies, ensuring that the findings synthesized in the systematic review are built upon a foundation of methodologically sound and reliable research.

### Data extraction and analysis

The extracted data was recorded in two separate forms. The first form included general characteristics of the article, such as the article's identification number in this study, title of the study, first author of the article, year of the study, type of study, country or city where the study was conducted, the subject matter extracted from the article for the present review, and the study's objective. The second form was related to the extraction of definitions and features of climate-resilient cities. Then, descriptive and thematic analysis was performed for the selected articles and texts. In this study, the authors coded the extracted descriptive information, definitions, and features of climate-resilient cities. Then, similar codes were grouped together. Finally, the grouped findings were analyzed for categorizing these strategies. The accuracy and completeness of the extracted data were discussed by the research team in a group discussion.

## Results

During the research, in the first stage of the main review study, 6,104 articles and documents were identified through the search process. After removing 903 cases of duplicate articles, 5,201 articles remained and were reviewed and screened. In the second stage, after studying the titles and abstracts, 4,176 articles were excluded from the retrieved studies. Finally, the full-text of 1,025 articles was reviewed, and based on the inclusion and exclusion criteria, 987 articles were removed. In the end, 38 studies related to the definitions and features of climate-resilient cities were included in the main review study and were examined and analyzed. The results of evaluating the entered articles with the CASP tool showed that 70% of the studies had high quality, and 30% had moderate quality (Table 1) (Fig. 1 provides a quick overview of how data was collected).

**Table 1:** Characteristics of the studies included in the review of definitions and characteristics of climate-adaptive cities

**NO**	**Title**	**1st Author (Year)**	**Country/ city**	**Type of study**	**Extracted subject**	**Study objective**	**Appraised Quality**
1	In pursuit of resilient, low carbon communities: An examination of barriers to action in three Canadian cities	Burch, S. (2010) [27]	Canada/the Lower Mainland of British Columbia:North Vancouver Vancouver Delta/	Original	Definition of Adaptation to climate change	(1) Providing organizational and socio-cultural barriers by collecting perspectives from interdisciplinary texts; (2) Investigating the effective barriers to utilizing various capacities for achieving greenhouse gas reduction and resilience in the three studied communities; and (3) Understanding dynamic interactions and relative importance of these barriers at the local level.	Moderate
2	Windows of opportunity: Addressing climate uncertainty through adaptation plan implementation	Abunnasr Y et al. (2015) [28]	Lebanon/ Beirut	Review	Definition of Adaptation to climate change	An investigation of innovations in urban water systems.	Moderate
3	Understanding conceptual climate change meanings and preferences of multi-actor professional leadership in New York	Keenan JM et al. (2016) [29]	United States/New York/	Survey	Definition of Adaptation / Characteristics of a Climate-Adaptive City	The concept of climate change / Conceptual evaluation of climate change meanings among various professional leaders.	High
4	Developing adaptive capacity in times of climate change in central rural Vietnam: exploring smallholders' learning and governance	Le TH. (2017) [30]	Central rural Vietnam/Vietnam	Dissertation	Definition of Adaptation / Characteristics of a Climate-Adaptive City	Capacity building for climate adaptation.	High
5	Adaptation to Climate Change: From Resilience to Transformation	Mark Pelling. (2010) [31]	United Kingdom / London/ Routledge	Book	Definition of Adaptation	Comprehensive analysis of the social dimensions of climate change adaptation.	High
6	Biophysical metrics for detecting more sustainable urban forms at the global scale	Cochran and Brunsell. (2017) [32]	United States/ Kansas	Case study	Definition of a Climate-Adaptive City	The aim of this study is to assess the feasibility of utilizing 1-km MODIS satellite data as a quick and straightforward means to evaluate urban sustainability worldwide, using any urban classification system. This data is easily accessible and provides a higher temporal resolution, requiring less processing time compared to remote sensing products with higher spatial resolution.	High
7	Extreme sea-level rise and adaptation options for coastal resort cities: A qualitative assessment from the Gold Coast, Australia	Cooper and Lemckert. (2012) [33]	Australia/Gold Coast/	Original	Definition of Adaptation	A qualitative assessment of adaptation strategies for sea-level rise in coastal resort towns, based on lessons learned from coastal management practices, current observations, and discussions with local stakeholders	Moderate
8	Experiencing and responding to everyday weather in Darwin, Australia: The important role of tolerance.	Eliza de Vet. (2017) [34]	Australia/Darwin	Original	Definition of Adaptation	An investigation into the role of air in daily life in the hot and humid region of Darwin, with a focus on determining participants' enthusiasm for connecting with air regardless of challenging weather conditions and access to air conditioning devices.	Moderate
9	Urban Design and Planning in Adapting to Climate Change: Advances, Applications, and Challenges.	Dhar TK. (2016) [35]	Jamaica (Caribbean Small Island Developing State)/Long Bay, Negril	Thesis	Definition of Adaptation / Characteristics of a Climate-Adaptive City	What climate risks do indigenous people and their assets face? How can indigenous communities adapt to these risks? What indicators exist to assess the current resilience of the constructed Negril region? What alternative (indigenous or expert-based) approaches can be considered for greater adaptation?	High
10	Urban flood impact assessment: A state-of-the-art review	Hammond MJ et al. (2015) [36]	United Kingdom	Review	Definition of Adaptation	The aim is to quantitatively assess the cost-effectiveness of resilience measures and integrated flood management plans for different scenarios, including urban development, socio-economic trends, and climate change, with the goal of enhancing their adaptability.	Moderate
11	Understanding the systemic nature of cities to improve health and climate change mitigation	Chapman, R et al. (2016) [37]	Australia/New Zealand/	Case study	Definition of a Climate-Adaptive City	Describing the characteristics of urban systems and how key components interact with each other, and introducing a framework for identifying key elements in dynamic urban systems.	High
12	Building resiliency: A cross-sectional study examining relationships among health-related quality of life, well-being, and disaster preparedness	Gowan ME et al. (2014) [38]	Australia/New Zealand	Cross-sectional study	Definition of Adaptation	Assessing the characteristics of health-promoting behaviors that lead to changes in attitudes and protective health behaviors.	Moderate
13	Building up resilience in cities worldwide – Rotterdam as participant in the 100 Resilient Cities Programme	Spaans M and Waterhout B. (2016) [39]	Netherlands/Rotterdam	Viewpoint	Definition of a Climate-Adaptive City	Examining the strategies and frameworks for developing urban resilience in 100 resilient cities	Moderate
14	Combining analytical frameworks to assess livelihood vulnerability to climate change and analyse adaptation options	Reed MS et al. (2013) [40]	United Kingdom	Analytical research	Definition of a Climate-Adaptive City	Integrating theories of sustainable livelihoods with other analytical frameworks (such as ecosystem services, diffusion theory, social learning, adaptive management, and transition management) to assess the vulnerability of rural livelihoods to climate change	Moderate
15	Hurricane Sandy and adaptation pathways in New York: Lessons from a first-responder city.	Rosenzweig C and Solecki W. (2014) [41]	United states/New York	Case study	Definition of Resilience	This study examines how organizations in a city integrate with unified municipal adaptation strategies and evaluates the compatibility of the strategies with the impacts of Hurricane Sandy, which occurred in October 2012 in New York City, as a case study.	Moderate
16	Civil society organizations and adaptation to the health effects of climate change in Canada.	Poutiainen C et al. (2013) [42]	Canada	Systematic review	Definition of Adaptation	Identification and examination of compatibilities by civil society organizations for adapting to the health effects of climate change based on a systematic review of 190 organizational activities and 1,196 adaptation action reports."	High
17	Climate change adaptation policies and plans: A survey in 11 South East European countries.	Pietrapertosa F et al. (2018) [43]	Austria, Bosnia and Herzegovina, Bulgaria, Croatia, Greece, Hungary, Italy, Romania, Serbia, Former Yugoslav Republic of Macedonia, Ukraine	Survey	Definition of Adaptation	The initiatives for adaptation in 11 South European countries participating in the SEE OrientGate project are summarized, referring to the EU-supported policies and actions and their implementation at the national level.	High
18	Briefing: Adapting to a changing climate	Henderson K. (2009) [44]	United Kingdom/ London	Original	Definition of Adaptation	A case study on climate change adaptation, focusing on examples of best practices in local authorities in Europe, the EU-funded project for green and blue space adaptation in urban and eco-district areas.	High
19	Impacts of climate change on the municipal water management system in the Kingdom of Bahrain: Vulnerability assessment and adaptation options.	Al-Zubari WK et al. (2018) [45]	Bahrain	Original	Characteristics of a Climate-Adaptive City	Assessment of vulnerability and adaptation options.	High
20	Energy Efficiency and Global Warming Potential in the Residential Sector: Comparative Evaluation of Canada and Saudi Arabia.	AlHashmi M et al. (2017) [46]	Canada and Saudi Arabia	Technical Papers	Characteristics of a Climate-Adaptive City	Comparison of energy efficiency and global warming assessment in the residential sector of Canada and Saudi Arabia.	High
21	Sustainable development of energy, water and environment systems index for Southeast European cities.	Kilkis S. (2016) [47]	Europe/European Cities	Original	Characteristics of a Climate-Adaptive City	Developing a sustainability index for energy, water, and environmental systems.	Moderate
22	Decarbonization action plans using hybrid modeling for a low-carbon society: The case of Bangkok Metropolitan Area	Ali G. (2017) [48]	Thailand/Bangkok/	Original	Characteristics of a Climate-Adaptive City	Proposing carbon reduction action plans for the Greater Bangkok area.	High
23	Risk-Based Performance Assessment of Stormwater Drainage Networks under Climate Change: A Case Study in the City of Kingston	Nanos M. G and Filion Y. (2016) [49]	Jamaica/Kingston/	Case study	Characteristics of a Climate-Adaptive City	Evaluation of stormwater drainage networks under the influence of climate change	Moderate
24	Participative future scenarios for integrated coastal zone management	Carrero R et al. (2013) [50]	Ayamonte, South Western Spain/Spain/	Original	Characteristics of a Climate-Adaptive City	Identifying collaborative future scenarios for coastal zone management	Moderate
25	Urban design principles for flood resilience: Learning from the ecological wisdom of living with floods in the Vietnamese Mekong Delta	Liao KH et al. (2016) [51]	China/Hong Kong	Original	Characteristics of a Climate-Adaptive City	Flood resilience.	Moderate
26	Adaptability of Design of Residential Houses in Tabriz and Baku with the Native Culture and Climate	Abdolhoseyni J. (2011) [52]	Iran /Tabriz	Descriptive	Characteristics of a Climate-Adaptive City	Determining the transformation of urban residential building structures influenced by local culture and climate.	Moderate
27	Disaster Resilience of Critical Water Infrastructure Systems.	Matthews J C. (2015) [53]	United States	Technical Notes	Characteristics of a Climate-Adaptive City	Assessing disaster resilience of critical water infrastructure systems.	High
28	Mainstreaming urban climate resilience into policy and planning; reflections from Asia.	Friend R et al. (2014) [54]	Thailand/ Bangkok	Original	Characteristics of a Climate-Adaptive City	Exploring gaps in the main process of climate resilience in Vietnam, Thailand, and Indonesia.	Moderate
29	Developing an integrated water management strategy to overcome conflicts between urban growth, water infrastructure and environmental quality: A case study from Ashford, Kent.	Furey SG and Lutyens BC. (2008) [55]	UK/Ashford, Kent	Case study	Characteristics of a Climate-Adaptive City	Developing a comprehensive water management strategy to overcome conflicts between urban growth, water infrastructure, and environmental quality.	Moderate
30	Carbon emission allocation standards in China: A case study of Shanghai city	Gao G et al. (2015) [56]	China/Shanghai	Case study	Characteristics of a Climate-Adaptive City	Developing carbon allocation emission standards	High
31	Sub-region (district) and sector level SO2 and NOx emissions for India: assessment of inventories and mitigation flexibility	Garg A et al. (2001) [57]	India	Original	Characteristics of a Climate-Adaptive City	Providing a scenario for the emissions of total SO2 and NOx in India, including the trends of their emissions and sectoral shares, following emission estimations for each of the 466 regions of India (Indian Census, 1992) for the years 1990 and 1995, to identify the largest regions and sectors that can be targeted for emissions reduction.	Moderate
32	Decentralizing urban disaster risk management in a centralized system? Agendas, actors and contentions in Vietnam	Garschagen M. (2016) [58]	Germany	Original	Characteristics of a Climate-Adaptive City	Urban decentralization and risk management.	Moderate
33	Coping with storm surges on the Icelandic south coast: A case study of the Stokkseyri village	Geirsdóttir GE et al. (2014) [59]	Iceland/Stokkseyri/	Case study	Characteristics of a Climate-Adaptive City	Exploring the residents' perspectives in Stourbridge regarding flood events and qualitatively assessing their interpretation of community vulnerability, resilience, and adaptability to such events, and the resulting socio-economic impacts.	Moderate
34	An integrative regional resilience framework for the changing urban water paradigm	Gonzales P, and Ajami NK. (2017) [60]	United States/San Francisco Bay/	Case study	Characteristics of a Climate-Adaptive City	Presenting a bottom-up resilience framework based on social and organizational contexts for assessing various water resource strategies.	High
35	Preferences for sustainable, liveable and resilient neighbourhoods and homes: A case of Canberra, Australia. Sustainable cities and society.	Tapsuwan S et al. (2018) [61]	Australian/Canberra/	Original	Characteristics of a Climate-Adaptive City	Preparing a list of features for sustainable and resilient homes and evaluating people's priorities for these sustainability and resilience features.	High
36	Increases in the climate change adaption effectiveness and availability of vegetation across a coastal to desert climate gradient in metropolitan Los Angeles, CA, USA	Tayyebi A. and Jenerette GD. (2016) [62]	United States/ California	Original	Characteristics of a Climate-Adaptive City	Access to green space for effective urban adaptation, identifying diversity in the mutual relationships between green space coverage (Normalized Difference Vegetation Index), socio-economic status (neighborhood income), altitude, and land surface temperature (LST).	High
37	Anticipatory governance: A tool for climate change adaptation. Journal of the American Planning Association.	Quay R. (2010) [63]	United states/ Denver Water, New York City, and the City of Phoenix/	Review and Case study	Characteristics of a Climate-Adaptive City	Establishing the necessary foundations for effective climate change planning.	Moderate
38	Women's health Australia: What do we know? What do we need to know? Progress on the Australian longitudinal study of women's health 1995–2000.	Lee C. (2001) [64]	Australia	Book	Characteristics of a Climate-Adaptive City	The main objective of the project is to gain an understanding of the relationships between social roles, life events, and women's health, with the aim of establishing a foundation for enhancing health policies and services.	Moderate

### Descriptive analysis

The reviewed articles were primarily from the United States, accounting for approximately 18.42% of the total. Canada contributed around 7.89%, both individually and as part of a joint article with Saudi Arabia. Australia and England followed closely, with approximately 15.78% and 10.52%, respectively. Thailand comprised about 5.26% of the articles. Other countries collectively contributed approximately 42.13% of the total. Regarding the types of articles, original articles constituted the majority at around 39.4%. Case studies followed, representing about 21.05%, and review articles accounted for 7.8%. Additionally, there was one mixed review and case study, making up approximately 2.6% of the total. Furthermore, two books, two surveys, and two theses were identified, each contributing approximately 5% to the overall distribution.

51.3% of the reviewed articles referred to the characteristics of climate-adaptive cities, 2.3% to the definition of resilience, 20.6% to the definition of adaptation, 10.4% to the definition of climate-adaptive city, 7.7% to the definition of adaption to climate change, and 7.7% to both the definition and characteristics of climate-adaptive cities. The specifications of the entered articles are shown in Table 1.

### Thematic analysis

Given that this study focuses on defining and outlining the characteristics of climate-adaptive cities, the thematic analysis begins by separating codes related to the definitions of adaptive cities from those related to their features. Following this division, codes within each category are further classified into relevant subcategories. Both categories are integral to our understanding, as the definitions aim to articulate what constitutes an adaptive city, while the characteristics elaborate on the specific attributes of such cities. In essence, characteristics serve as complementary elements, providing detailed insights into the nature of adaptive cities.

A total of 75 codes were extracted from 38 articles, including 55 codes related to the characteristics of climate-resilient cities and 20 codes related to definitions. The highest number of codes in the category of features of climate-resilient cities was related to the subcategory of effective resource management with 18 codes. In the category of definitions, the highest number of codes was related to the definition of resilience with 16 codes (Table 2). In this review, only four definitions of climate-resilient cities were extracted. It should also be noted that the subcategories of stakeholder participation and knowledge utilization had the lowest number of codes with only three codes among the subcategories in this review. Based on this study, many of the features of climate-resilient cities were extracted from the experiences of resilient cities or cities that have taken steps in this regard.

**Table 2:** Reviewed categories and sub-categories of definitions, features and characteristics of adaptive cities to climate change

**Category**	**Sub- Category**	**Code**
**Definition of Climate Adaptation and Climate-Adaptive Cities**	**Climate Adaptation and Resilience**	• Adaptation capacity refers to the ability of stakeholders to organize themselves, develop knowledge, strengthen leadership, and make decentralized decisions [22]. • Adaptation capacity refers to the ability of individuals or groups to respond and adjust to environmental changes [22]. • Adaptation capacity refers to the ability of systems to adjust to climate change, such as coping with extreme weather conditions [22]. • Adaptation is a process through which stakeholders can reflect and respond to the impacts of changes in their operations, modify underlying infrastructure that creates risk, adjust their capacity to tolerate risks, and undertake other measures to adapt to climate change [23]. • Adaptation is the alignment between future climate change scenarios and strategies and plans for changing current practices [24]. • Adaptation is the capacity that a community demonstrates sustainably in response to environmental changes [25]. • Climate adaptation is the adjustment of natural or human systems to actual or potential climatic stimuli or their effects in order to moderate harm or exploit beneficial opportunities [26]. • Regarding flood resilience in urban environments, resilience can be defined as the capacity of a system, community, or society that is exposed to a hazard to resist, absorb, adapt, and recover from its effects in order to achieve an acceptable level of functioning and structure. In other words, resilience is equivalent to resistance, recovery, reflection, and response, which take into account the need to learn from the past [43]. • Resilience is the capacity of economic and social systems to sustain change and adapt within a critical life threshold [65]. • Adaptation is a practical response to public health, which is necessary to prevent, reduce, and manage climate change-related risks [33]. • Adaptation is a complex, place-based issue with multiple dimensions that is heavily dependent on climate, environmental, social, and political conditions [34]. • Adaptation means learning to live with more severe weather events and changing climate patterns, and preparing for other unavoidable changes [36]. • Climate adaptation is the "ability or potential of a system to successfully respond to climate vulnerability and change" [30]. • Climate adaptation refers to an action taken to prevent or reduce vulnerability to climate change impacts [44]. • Climate adaptation is the ability to respond to environmental changes, meaning maintaining the essential and minimum functioning of society against external stimuli [42]. • Climate resilience is a capacity of a system to dynamically adapt through risk reduction management against disasters [35].
	**Climate-Adaptive Cities**	• A climate-adaptive city is a city that can sustainably withstand the urban heat island effect [28]. • A climate-adaptive city is a city that applies change and mitigation solutions in a timely manner, before changes become unmanageable, and has learned from non-adaptive approaches taken by other cities [37]. • Urban resilience refers to the capacity of individuals, communities, institutions, businesses, and systems within a city to survive, adapt, and grow regardless of the type of chronic stresses and acute shocks experienced [39]. • A climate-adaptive city/society is one that has adapted from the bottom up, resulting in reduced social vulnerability [32].
**Characteristics of Climate-Adapted Cities**	**Stakeholder Participation**	• The importance of stakeholder engagement in achieving proper water resource management [66]. • Public participation/civic engagement [67]. • The effective role of social coordination in resilience [68].
	**Effective Resource Management**	• Effective resource management [66]. • Developing sustainable strategies by estimating and assessing each household's contribution to global warming based on different lifestyles and climatic conditions in different parts of the world [66]. • Reducing energy consumption to control greenhouse gas emissions [66]. • Greater attention to cost-effectiveness in reducing emissions of greenhouse gases such as SO2 and NOx [59]. • Effective energy management and utilization through the design of wind catchers, chimneys, special summer spaces with domed or elevated ceilings, courtyards, cellars, basements, underground water reservoirs, and natural refrigerators [50]. • Using new strategies for water and wastewater systems and flood control to enhance their resilience to climate change [45]. • Effective use of limited resources [22]. • Sustainability, resource management, and proper resource utilization [54]. • Resource management for achieving adaptation [54]. • Lifestyle changes and proper resource utilization [22]. • Capacity building and effective adaptation methods utilization [22]. • The effective role of better economic conditions in resilience of communities [68]. • Low-carbon technology and the provision of new sources of energy [69]. • Sustainable centralized water management to overcome challenges related to urban development, water infrastructure, and environmental quality [47]. • The impact of access to vegetation cover on effective adaptation [70].
	**Foresight in Planning**	• Foresight in planning [71]. • Developing scenario-based water adaptation planning [45]. • Reducing the use of fossil fuels and utilizing renewable energy sources, such as solar, wind, nuclear, and bioenergy, to reduce carbon footprint [57]. • Foresight and future planning to achieve adaptation by predicting future changes and developments [57]. • The necessity of adaptation and the development of executive criteria for assessing the flexibility and vulnerability of urban drainage systems, with predictions and modeling of practical measures for severe weather periods, in order to evaluate how a future storm will behave [63]. • Foresight for achieving adaptation [63]. • Considering the economic dimensions in climate change policy planning, identifying stakeholders and their participation, and looking to the future [52]. • Foresight, efficiency, and proper resource utilization as key factors in achieving a resilient city [52]. • A future-oriented and collectively compatible response to the situation [42]. • Attention to past flood experiences, household preparedness, resilient infrastructure design, and local capacity building [62].
	**Education**	• Education on adaptation methods at all levels [22]. • Expanding adaptation capacities [22]. • Utilizing the capacities of social media to increase adaptation capacity [22]. • Increasing the learning of local farmers to enhance their resilience to climate change [22]. • Learning management and adaptation strategies to cope with the effects of climate change [22]. • Community awareness of the conditions in which they live [68].
	**Utilizing Knowledge**	• Knowing and being able to recognize weather and sea change signs to prepare for responding to risks [68]. • Utilizing indigenous knowledge and community-based approaches, leveraging interdisciplinary knowledge and governmental cooperation, and integrating physical and socio-ecological characteristics to achieve successful adaptation [26]. • Utilizing knowledge, including indigenous knowledge and interdisciplinary research [26].
	**Innovation in Governance and Industry**	• The important role of governance in enhancing resilience [22]. • Innovation [69]. • Low-carbon development policies through industrial structure and innovation in governance [69]. • Incentive mechanisms for using low-carbon technologies [69]. • Increasing local water resource resilience through a bottom-up approach to decision-making [55]. • Low-energy consumption through sustainable house design [56]. • Attention to energy and carbon dioxide emissions, transportation system, waste management, water, socio-economic capacity, and intersectoral sustainability for achieving sustainable development in cities [54]. • Attention to resilience and capacity-building aspects [62].
	**Decentralized Climate Change Management**	• Increasing the capacity of local government to assist in the adaptation of the people, especially farmers, to environmental changes [22]. • Decentralized urban risk management [72]. • Distributed urban risk management [46]. • Adaptation to climate change requires decentralized planning based on local risk assessment [72]. • Increasing local water resource resilience through a bottom-up approach to decision-making [72].
	**Low-Carbon Economy**	• Low-carbon economy as one of the prominent characteristics of resilient cities [49]. • Creating green jobs [49]. • Transforming existing jobs into green jobs [49]. • Ability to continue working in more resource-efficient conditions [49]. • Attention to low-carbon economy [29].

## Discussion

**Fig. 2:**
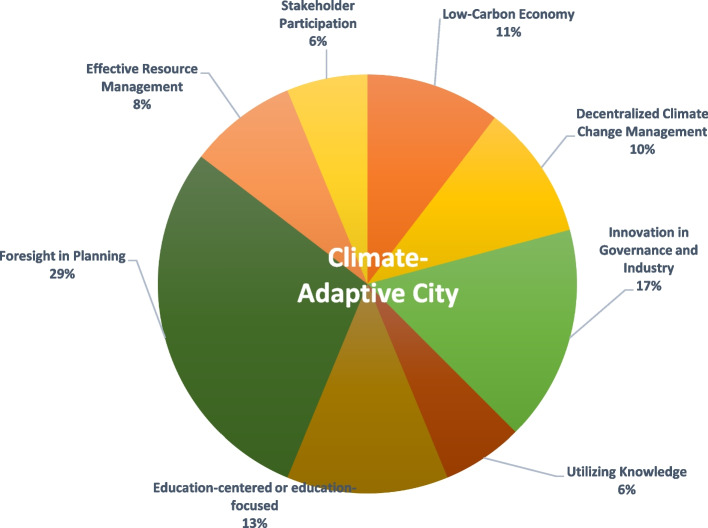
Characteristics and features of climate-adaptive cities identified through systematic review

In none of the reviewed studies, comprehensive definitions and features of climate-resilient cities were thoroughly investigated. Introducing a novel and comprehensive definition of climate-resilient cities, along with categorizing their features and characteristics, holds significant potential for contributing to the existing body of literature. This contribution extends to enhancing the resilience of cities in diverse regions worldwide. Moreover, delineating the features and characteristics of climate-resilient cities in this study proves to be highly efficacious in evaluating the resilience level of cities and urban areas to climate change, while concurrently pinpointing prevailing weaknesses and challenges (Fig. 2).

### Definition of climate adaptation and climate-adaptive cities

According to definitions of climate adaptation, it is a multidimensional, location-based challenge [43]. In fact, adaptation to climate change is a process [65], and in another definition, it is the coordination between future climate change scenarios and current change strategies and programs [33]. Some definitions of climate adaptation refer to capacities, which are sustainable and the abilities of communities to cope with environmental changes [34], or the capacity of a system, society, or community to resist or adapt to a risk, to achieve and maintain a level of performance and structure [36]. However, the capacity for adaptation is more interpreted as the ability of stakeholders, individuals, groups, and systems [30]. In another definition, climate adaptation is defined as learning to live with severe weather events, changing weather patterns, and preparing for some unavoidable changes [44]. From the health perspective, adaptation is a responsive action for public health, which is necessary to prevent, reduce, and manage climate-related risks [42]. In fact, climate adaptation is the adjustment of natural or human systems to real or potential climate stimuli or their effects, mitigating damages and taking advantage of opportunities [35] and results in preventing or reducing vulnerability to climate change [28].

Although there are numerous definitions regarding adaptation to climate change, the definitions of climate-adapted cities are limited and very few. Moreover, none of these definitions are comprehensive, applying solutions to change and reducing the effects in a timely and effective manner before uncontrollable changes occur, and learning from non-adaptive ways used by other cities for defining climate-adapted cities [37]. In this definition, solutions are generally mentioned, while clarifying this issue is very important. Another definition mainly refers to reducing social vulnerability and emphasizes the importance of bottom-up adaptation [39], while ignoring other characteristics of climate-adapted cities.

In another definition, a climate-adapted city is defined as a city that can only maintain stability against heat islands [32]. Although this definition is in line with the global Paris Agreement aimed at global adaptation, it cannot be considered a comprehensive goal. This goal seeks to ensure sufficient adaptation response to the global temperature goal, ultimately leading to sustainable development [20, 66]. Finally, a relatively better definition of urban resilience to climate change is as follows: the flexibility of the capacity of individuals, communities, institutions, businesses, and systems within a city to survive, adapt, and grow regardless of the chronic stress and acute shocks they experience [39]. Therefore, based on the characteristics of climate-adapted cities and the texts reviewed in this study, it can be suggested that a climate-adapted city is a city that, through effective resource management, future-oriented planning, education, knowledge utilization, innovation in governance and industry, decentralized management, and low-carbon economy, leads to adaptation, resilience, sustainability, and flexibility of the capacity of individuals, communities, institutions, businesses, and systems within a city, against all impacts of climate change and reducing the resulting consequences.

### Characteristics of climate-adapted cities

In the literature review conducted in this study, no specific study was found to investigate the characteristics of climate-adapted cities. Nevertheless, determining the features, characteristics, and standards of adaptation can be highly effective in assessing its efficiency and categorizing the factors that foster adaptive capacity.

Identifying adaptation criteria is often challenging for development interventions, which can lead to difficulties in classifying whether anything that creates adaptive capacity can be called adaptation [67, 68]. Therefore, in this review, all variables and factors that can be considered as characteristics of a climate-adapted city were extracted and categorized in a scientific and systematic manner.

### Stakeholder participation

Based on the literature review and considering the effective role of social coordination in resilience [59], one of the characteristics of climate-adapted cities is the use of participatory techniques, including local stakeholders [50] and community participation [45, 54]. Friend (2010) also considers community participation as a prominent feature of climate-adapted cities [54]. Although this participation should be comprehensive, Al-Zubari et al. (2018) only referred to the necessity of stakeholder participation in achieving proper water resources [45]. However, the community-based adaptation process in climate-adapted cities involves engaging other institutional elements in decision-making, ensuring the compatibility of top-down planning with local needs, and using participatory research to facilitate the participation of local communities in shaping adaptation planning processes [21]. Therefore, it can be said that community-based adaptation can provide an opportunity for people's participation in planning and adaptation activities in a comprehensive and proper manner. Facilitating people's participation leads to understanding and enhancing their awareness of their risk, vulnerability, and resilience to climate change [69].

### Effective resource management

Effective resource management [45, 47] and efficient use of limited resources [30, 47] are among the most important characteristics of climate-adapted cities. Al-Zubari (2018) [45] emphasizes the need to create sustainable strategies by estimating the assessment of each household's contribution to global warming based on different lifestyles and climatic conditions in different parts of the world, as well as reducing energy consumption to control greenhouse gas emissions [45]. This highlights the importance of resource management for achieving effective adaptation. This adaptation strategy may vary across different climates, as individuals' thermal responses to a consistent thermal environment differ from one location to another [70].

Kilkis S (2016) [47] also highlights the importance of sustainability, resource management, and the judicious use of resources in climate-adapted cities. He argues that achieving sustainable development in cities requires attention to factors such as energy consumption, carbon dioxide emissions, transportation systems, waste management, water resources, socio-economic capacity, and inter-sectoral sustainability [47]. Although, the current global progress and sustainable initiatives, as outlined in international frameworks such as the Paris Climate Agreement and the United Nations Sustainable Development Goals (SDGs), are not advancing rapidly or at the expected pace in reducing greenhouse gas emissions and addressing climate change. In other words, advancements in these areas are encountering challenges and obstacle [71].

In line with the focus on transportation systems, Garg (2001) writes in his article that since greenhouse gases SO2 and NOx in Indian cities are more emitted from industries, focusing on transportation systems as sources of greenhouse gas emissions will have a higher cost-effectiveness in reducing emissions [57]. Based on the above, it can be concluded that reducing greenhouse gas emissions from urban transportation systems is one of the characteristics of climate-adapted cities, which will also lead to economic benefits.

In his study, Quay R (2010) presents the characteristics of different cities based on a review of their experiences. For example, the city of Denver has developed a water adaptation planning process based on scenarios, while New York City has developed new strategies for water and sewage systems and flood control to enhance their resilience to climate change [63]. In addition to these, other solutions for effective resource management towards climate adaptation have been mentioned, including: effective energy use management through wind catchers, chimneys, summer spaces with dome or elevated ceilings, courtyards, basements, underground water tanks, and natural refrigerators [52]; access to vegetation coverage [62]; centralized sustainable water management [55]; lifestyle change and proper use of resources [30]; low-carbon technologies and new energy sources [56]; focusing on cost-effectiveness in reducing greenhouse gas emissions [57]; reducing energy consumption to control greenhouse gas emissions [45]; and the use of renewable energy sources [72] for climate adaptation in urban areas.

### Foresight in planning

One of the most important characteristics of climate-adapted cities is foresight, future prediction, and planning for the future to achieve effective adaptation. In this regard, Alhashmi et al. (2017) emphasizes the need for planning to reduce the use of fossil fuels and use renewable energy sources such as solar, wind, nuclear, and biomass to reduce carbon emissions [46]. Nanos and Filion (2016) also point to the importance of foresight in climate adaptation planning, considering resilience and executive criteria to assess the flexibility and vulnerability of urban drainage networks in Kingstone. They argue that this can be achieved through forecasting and designing models for hard weather periods to assess how a storm will behave in the future [49]. Carrero et al. (2013) also mentions the characteristics of foresight, stakeholder participation, and resource management. In his article, he emphasizes considering the economic dimensions in climate change policy planning, identifying stakeholders and their participation, and having a future-oriented approach as effective factors for management [50]. Additionally, Keenan et al. (2016) includes foresight and collective response to current conditions as characteristics of resilience [29]. Therefore, foresight, resource efficiency, and proper resource management can be considered essential for cities to achieve climate adaptation.

### Education

One of the key characteristics of climate-adaptive cities is education, which involves teaching adaptation strategies at all levels. THP (2017) suggests using social media to increase local farmers' adaptability and learning to enhance resilience to climate change. Therefore, it can be concluded that education and capacity building on effective adaptation strategies and changes in lifestyle and resource use are critical for climate adaptation [30]. Geirsdóttir et al. (2014) emphasizes the importance of community awareness of their living conditions in climate adaptation. For example, knowledge of past communities in reading weather signs and sea changes had prepared them to react to hazards and raised their preparedness level [59]. In conclusion, numerous adaptation strategies and solutions can be taught to communities to enhance their participation in climate adaptation. Therefore, one of the characteristics of climate-adaptive communities is to pay attention to the following while teaching adaptation strategies: using learning methods and teaching techniques appropriate for the community's culture and awareness level, using social media capacity, using virtual spaces and modern teaching methods, and engaging local leaders and educators for education.

### Utilizing knowledge

The gap between knowledge and action in the field of climate change has made it difficult to understand and establish a relationship with it [73]. Tapan Kumar Dhar (2016) [35] believes that using indigenous, interdisciplinary and community-based knowledge along with governmental collaborations, as well as integrating physical and socio-environmental characteristics, are necessary for successful adaptation [35]. Therefore, it seems that the use of indigenous knowledge and interdisciplinary research [35], is one of the necessities for achieving climate-adaptive cities. Community awareness of their living conditions can play a significant role in identifying and interpreting environmental changes, which can enhance their preparedness and response to climate change hazards [59]. In this regard, Odemerho emphasizes the importance of utilizing the experiences and human knowledge of flood-prone areas to adapt to floods and recognize the dominant type of flood and its root causes [74, 75]. Overall, it can be inferred that utilizing knowledge, especially interdisciplinary, local, and indigenous knowledge, and utilizing past experiences are characteristics of climate-adaptive cities and can enhance their awareness, preparedness, and adaptation to climate change.

### Innovation in governance and industry

Governance [30] and industry play a crucial role in enhancing the resilience of communities to climate change, and innovation in these areas is one of the key characteristics of climate-adaptive cities. For example, Guangkuo Gao (2015) [56] considers the development of low-carbon policies through industrial structure, innovation in governance, low-carbon technology, incentive mechanisms, and new energy supply as characteristics of climate-adaptive cities [56]. Based on the review conducted in this study, other examples of innovation in governance and industry include: increasing the resilience of local water resources with a bottom-up approach in decision-making [60], low-energy consumption through sustainable house design [61], attention to energy and carbon dioxide emissions, transportation systems, waste management, water, social-economic capacity, and intersectoral sustainability for achieving sustainable development in cities [47], and attention to resistance and capacity building [51]. Therefore, the adaptation of climate-adaptive cities requires the creation, expansion, or imitation of innovative strategies and plans for climate adaptation in governance and industry. This issue, including some insights on corporate social responsibility, should receive the attention and support of policymakers and industry leaders.

### Decentralized climate change management

Another characteristic of climate-adaptive cities is decentralized management. One example of this is decentralized urban risk management [53, 58]. For instance, decentralization of urban risk management in the central system in Vietnam is an example of decentralization [53]. One aspect of climate adaptation is decentralized planning based on local risk assessments. Moreover, decentralized management signifies the involvement and participation of communities in decision-making. In this regard, Gonzales (2017) argues that a bottom-up approach in decision-making can help increase the resilience of local water resources [60]. Furthermore, increasing the capacity of local governments to assist the adaptive growth of people, especially farmers, to environmental changes has been recommended [30].

### Low-carbon economy

One of the prominent features of climate-adaptive cities is a focus on a low-carbon economy [64]. Li (1995) identifies the creation of green jobs, the possibility of transforming existing jobs into green jobs, and the ability to continue working under lower consumption conditions as examples of a low-carbon economy [76]. In this regard, it is also possible to mention the imposition of taxes on carbon dioxide, its trading, and investment in wind, solar, water, biomass, and other types of renewable energy [56] should be promoted. Although recent studies on the transition to a global low-carbon economy or decarbonization are not encouraging, as both human and natural carbon dioxide emissions are increasing due to human factors [77], attention to this issue is essential for climate-adaptive cities. In summary, a low-carbon economy is critical for reducing greenhouse gas emissions and addressing climate change. Climate-adaptive cities must prioritize the development of a low-carbon economy to reduce their carbon footprint and promote sustainability.

The main limitations of this study were associated with the extended duration of the project. This study was part of a larger systematic review. Due to the substantial scale of the overarching project, the execution time of the work and its completion extended. While the implementation date is specified in the methodology, the prolonged duration can be justified to some extent given the significance of the climate change issue and the contemporary nature of the topic. Another limitation pertained to accessing articles. Some articles were not readily available, prompting researchers to attempt retrieval through contacting authors, purchasing articles, or utilizing accessible academic databases.

## Conclusion

Based on the definitions and characteristics examined in this systematic review, a climate-adaptive city is a city that, through effective resource management, forward-thinking planning, education, knowledge utilization, innovation in governance and industry, decentralized management, and low-carbon economy, can adapt, be resilient, sustainable, and flexible in the face of all possible climate change impacts and minimize their negative consequences on the capacity of individuals, communities, institutions, businesses, and systems within a city. It should be noted that all actions must be in line with the economic, social, cultural, and geographical characteristics of each region separately and must be based on sustainable development.

Forward-thinking planning in this regard must be community-based and resource management with a bottom-up approach in decision-making. In a climate-adaptive city, the participation of all stakeholders and local communities must be facilitated in a way that ultimately leads to reduced social vulnerability and economic efficiency.

Conclusively, future research in this field should prioritize the issue of carbon justice, a pivotal element in achieving sustainability and resilience in climate-adaptive cities. Additionally, we recommend conducting foundational studies to thoroughly explore decision-makers' attitudes, contributing to the development of appropriate protocols, principles, and urban plans. Subsequent research can extensively investigate the roles of corporate entities, academia, and industries in climate-adaptive city development. In conclusion, this study underscores the urgent need for a more comprehensive approach to climate change adaptation in urban planning.

## Data Availability

The datasets used and/or analyzed during the current study are available from the corresponding author on reasonable request.

## References

[1] Rosenzweig C, Solecki WD, Hammer SA, Mehrotra S. Climate change and cities: First assessment report of the urban climate change research network. United States of America, New York: Cambridge University Press; 2011.

[2] Sharifi A (2021). Co-benefits and synergies between urban climate change mitigation and adaptation measures: a literature review. Sci Total Environ.

[3] Alabsi AAN, Wu Y, Koko AF, Alshareem KM, Hamed R (2021). Towards climate adaptation in cities: indicators of the sustainable climate-adaptive urban fabric of traditional cities in West Asia. Appl Sci.

[4] Singh C, Madhavan M, Arvind J, Bazaz A (2021). Climate change adaptation in Indian cities: a review of existing actions and spaces for triple wins. Urban Climate.

[5] Almusaed A, Almssad A, Homod RZ, Yitmen I (2020). Environmental profile on building material passports for hot climates. Sustainability.

[6] Zhang J, Khoshbakht M, Liu J, Gou Z, Xiong J, Jiang M (2022). A clustering review of vegetation-indicating parameters in urban thermal environment studies towards various factors. J Therm Biol.

[7] Organization WM. Causes of climate change Geneva: World Health Organization 2009 Agust. 2009 Agust. Available from: Available from: https://www.ipcc.ch/.

[8] Hughes TP, Baird AH, Bellwood DR, Card M, Connolly SR, Folke C (2003). Climate change, human impacts, and the resilience of coral reefs. Science.

[9] Portier CJ, Tart KT, Carter SR, Dilworth CH, Grambsch AE, Gohlke J (2013). A human health perspective on climate change: a report outlining the research needs on the human health effects of climate change. J Curr Issues Global.

[10] World Health Organization. Protecting health from climate change: connecting science, policy and people. 2009.

[11] Heaviside C, Macintyre H, Vardoulakis S (2017). The urban heat island: implications for health in a changing environment. Curr Environ Health Rep.

[12] Dasgupta S, Laplante B, Murray S, Wheeler D (2011). Exposure of developing countries to sea-level rise and storm surges. Clim Change.

[13] Adger WN, Lorenzoni I, O'Brien KL. Adapting to climate change: Thresholds, values, governance. United States of America, New York: Cambridge University Press; 2009.

[14] Glickman TS (2000). Glossary of meteorology: American Meteorological Soc.

[15] Salehi S, Ardalan A, Garmaroudi G, Ostadtaghizadeh A, Rahimiforoushani A, Zareiyan A. Climate change adaptation: a systematic review on domains and indicators. Natural Hazards. 2019;96:521-50.

[16] Friel S, Bowen K, Campbell-Lendrum D, Frumkin H, McMichael AJ, Rasanathan K (2011). Climate change, noncommunicable diseases, and development: the relationships and common policy opportunities. Annu Rev Public Health.

[17] McMichael AJ C-LD, Corvalán CF, Ebi KL, Githeko A, Scheraga JD, et al. Climate change and human health: risks and responses. Geneva: World Health Organization; 2003.

[18] Mobarghaei N, Mokhtari Z (2018). Applying ecosystem-based adaptation approach in building climate-resilient cities. Popularization Sci.

[19] Kou Y, Xian D, Liu Y, Chen J, Wang C, Cheng B (2022). Factors affecting urban climate at different times of the day in China: a case study in Yibin, a riverside mountain city. Nat Based Solutions.

[20] Singh C, Iyer S, New MG, Few R, Kuchimanchi B, Segnon AC (2022). Interrogating ‘effectiveness’ in climate change adaptation: 11 guiding principles for adaptation research and practice. Climate Dev.

[21] Archer D, Almansi F, DiGregorio M, Roberts D, Sharma D, Syam D (2014). Moving towards inclusive urban adaptation: approaches to integrating community-based adaptation to climate change at city and national scale. Climate Dev.

[22] Zhang J, Gou Z, Cheng B, Khoshbakht M (2022). A study of physical factors influencing park cooling intensities and their effects in different time of the day. J Therm Biol.

[23] Campbell-Lendrum D, Corvalan C (2007). Climate change and developing-country cities: implications for environmental health and equity. J Urban Health.

[24] Reckien D, Flacke J, Olazabal M, Heidrich O (2015). The influence of drivers and barriers on urban adaptation and mitigation plans—an empirical analysis of European cities. PLoS ONE.

[25] Jung S, Uttley L, Huang J (2022). Housing with care for older people: a scoping review using the CASP assessment tool to inform optimal design. HERD: Health Environ Res Design J.

[26] Monaghesh E, Hajizadeh A (2020). The role of telehealth during COVID-19 outbreak: a systematic review based on current evidence. BMC Public Health.

[27] Burch S. In pursuit of resilient, low carbon communities: An examination of barriers to action in three Canadian cities. Energy Policy. 2010;38(12):7575-85.

[28] Abunnasr Y, Hamin EM, Brabec E (2015). Windows of opportunity: Addressing climate uncertainty through adaptation plan implementation. J Environ Planning Manage.

[29] Keenan JM, King DA, Willis D (2016). Understanding conceptual climate change meanings and preferences of multi-actor professional leadership in New York. J Environ Plan Policy Manag.

[30] Le THP. Developing adaptive capacity in times of climate change in central rural Vietnam: exploring smallholders’ learning and governance: Wageningen University; 2017.

[31] Pelling M. Adaptation to climate change: from resilience to transformation. Routledge; 2010.

[32] Cochran FV, Brunsell NA (2017). Biophysical metrics for detecting more sustainable urban forms at the global scale. Int J Sustain Built Environ.

[33] Cooper J, Lemckert C (2012). Extreme sea-level rise and adaptation options for coastal resort cities: a qualitative assessment from the Gold Coast. Australia Ocean Coastal Manag.

[34] De Vet E (2017). Experiencing and responding to everyday weather in Darwin, Australia: the important role of tolerance. Weather Climate Soc.

[35] Dhar TK (2016). Urban design and planning in adapting to climate change: Advances, applications, and challenges.

[36] Hammond MJ, Chen AS, Djordjević S, Butler D, Mark O (2015). Urban flood impact assessment: a state-of-the-art review. Urban Water Journal.

[37] Chapman R, Howden-Chapman P, Capon A (2016). Understanding the systemic nature of cities to improve health and climate change mitigation. Environ Int.

[38] Gowan ME, Kirk RC, Sloan JA. Building resiliency: a cross-sectional study examining relationships among health-related quality of life, well-being, and disaster preparedness. Health and quality of life outcomes. 2014;12:1-17. 10.1186/1477-7525-12-85 PMC4062284 24909780

[39] Spaans M, Waterhout B (2017). Building up resilience in cities worldwide–Rotterdam as participant in the 100 resilient cities programme. Cities.

[40] Reed MS, Podesta G, Fazey I, Geeson N, Hessel R, Hubacek K, et al. Combining analytical frameworks to assess livelihood vulnerability to climate change and analyse adaptation options. Ecological Economics. 2013;94:66-77. 10.1016/j.ecolecon.2013.07.007 PMC4375565 25844020

[41] Rosenzweig C, Solecki W. Hurricane Sandy and adaptation pathways in New York: Lessons from a first-responder city. Global Environmental Change. 2014;28:395-408.

[42] Poutiainen C, Berrang-Ford L, Ford J, Heymann J (2013). Civil society organizations and adaptation to the health effects of climate change in Canada. Public Health.

[43] Pietrapertosa F, Khokhlov V, Salvia M, Cosmi C (2018). Climate change adaptation policies and plans: a survey in 11 South East European countries. Renew Sustain Energy Rev.

[44] Henderson K (2010). Briefing: adapting to a changing climate. Proceed Institution Civil Engineers-Urban Design Planning.

[45] Al-Zubari WK, El-Sadek AA, Al-Aradi MJ, Al-Mahal HA (2018). Impacts of climate change on the municipal water management system in the Kingdom of Bahrain: vulnerability assessment and adaptation options. Clim Risk Manag.

[46] AlHashmi M, Haider H, Hewage K, Sadiq R (2017). Energy efficiency and global warming potential in the residential sector: comparative evaluation of Canada and Saudi Arabia. J Archit Eng.

[47] Kilkis S. Sustainable development of energy, water and environment systems index for Southeast European cities. J Clean Prod. 2016;130(1):222-34.

[48] Ali G, Pumijumnong N, Cui S. Decarbonization action plans using hybrid modeling for a low-carbon society: the case of Bangkok Metropolitan Area. J Clean Prod. 2017;168:940-51.

[49] Nanos MG, Filion Y (2016). editors. risk-based performance assessment of stormwater drainage networks under climate change: a case study in the City of Kingston, ON. World Environ Water Res Congress.

[50] Carrero R, Navas F, Malvárez G, Cáceres F (2013). Participative future scenarios for integrated coastal zone management. J Coastal Res.

[51] Liao K-H, Le TA, Van Nguyen K (2016). Urban design principles for flood resilience: learning from the ecological wisdom of living with floods in the Vietnamese Mekong Delta. Landsc Urban Plan.

[52] Abdolhoseyni J (2011). Adaptability of design of residential houses in Tabriz and Baku with the native culture and climate. Monthly Sci J Bagh-e Nazar.

[53] Matthews J (2015). Disaster Resilience of critical water infrastructure systems. J Struct Eng.

[54] Friend R, Jarvie J, Reed SO, Sutarto R, Thinphanga P, Toan VC (2014). Mainstreaming urban climate resilience into policy and planning; reflections from Asia. Urban Climate.

[55] Furey S, Lutyens B (2008). Developing an integrated water management strategy to overcome conflicts between urban growth, water infrastructure and environmental quality: a case study from Ashford. Kent Water Environ J.

[56] Gao G, Chen S, Yang J (2015). Carbon emission allocation standards in China: a case study of Shanghai city. Energ Strat Rev.

[57] Garg A, Shukla P, Bhattacharya S, Dadhwal V (2001). Sub-region (district) and sector level SO2 and NOx emissions for India: assessment of inventories and mitigation flexibility. Atmos Environ.

[58] Garschagen M (2016). Decentralizing urban disaster risk management in a centralized system? Agendas, actors and contentions in Vietnam. Habitat Int.

[59] Geirsdóttir GE, Gísladóttir G, Jónsdóttir Á (2014). Coping with storm surges on the Icelandic south coast: A case study of the Stokkseyri village. Ocean Coast Manag.

[60] Gonzales P, Ajami NK (2017). An integrative regional resilience framework for the changing urban water paradigm. Sustain Cities Soc.

[61] Tapsuwan S, Mathot C, Walker I, Barnett G (2018). Preferences for sustainable, liveable and resilient neighbourhoods and homes: a case of Canberra. Australia Sustainable Cities Soc.

[62] Tayyebi A, Jenerette GD (2016). Increases in the climate change adaption effectiveness and availability of vegetation across a coastal to desert climate gradient in metropolitan Los Angeles, CA, USA. Sci Total Environ.

[63] Quay R (2010). Anticipatory governance: A tool for climate change adaptation. J Am Plann Assoc.

[64] Lee C (2001). What do we know? What do we need to know? Women's Health Australia: Progress on the Australian longitudinal study on women's health, 1995− 2000.

[65] Peling SWY. Perbedaan Pengaruh Metode Latihan Beban Leg-Press dan Sqat terhadap Peningkatan Prestasi Lari 100 Meter Ditinjau dari Waktu Reaksi (Studi Eksperimen pada Mahasiswa Putra Pembinaan Prestasi Atletik Fakultas Olahraga dan Kesehatan Universitas Pendidikan: UNS (Sebelas Maret University). 2011.

[66] Craft B, Fisher S (2018). Measuring the adaptation goal in the global stocktake of the Paris agreement. Climate Policy.

[67] Owen G (2020). What makes climate change adaptation effective? A systematic review of the literature. Glob Environ Chang.

[68] Schipper ELF, Tanner T, Dube OP, Adams K, Huq S (2020). The debate: Is global development adapting to climate change?. World DeveloP Perspect.

[69] Nirupama N, Maula A (2013). Engaging public for building resilient communities to reduce disaster impact. Nat Hazards.

[70] Zhang J, Guo W, Cheng B, Jiang L, Xu S (2022). A review of the impacts of climate factors on humans’ outdoor thermal perceptions. J Therm Biol.

[71] He B-J, Wang J, Zhu J, Qi J (2022). Beating the urban heat: Situation, background, impacts and the way forward in China. Renew Sustain Energy Rev.

[72] Sakr D, Baas L, El-Haggar S, Huisingh D (2011). Critical success and limiting factors for eco-industrial parks: global trends and Egyptian context. J Clean Prod.

[73] Mendizabal M, Feliu E, Tapia C, Rajaeifar MA, Tiwary A, Sepúlveda J (2021). Triggers of change to achieve sustainable, resilient, and adaptive cities. City Environ Interactions.

[74] Odemerho FO (2015). Building climate change resilience through bottom-up adaptation to flood risk in Warri. Nigeria Environment and Urbanization.

[75] Yari A, Ostadtaghizadeh A, Ardalan A, Zarezadeh Y, Rahimiforoushani A, Bidarpoor F. Risk factors of death from flood: Findings of a systematic review. Journal of environmental health science and engineering. 2020;18(2):1643-53. 10.1007/s40201-020-00511-x PMC7721754 33312668

[76] Lee C (2000). What do we know? What do we need to know. Women’s Health Australia: Progress on the Australian longitudinal study on women’s health.

[77] Lugo-Morin DR (2021). Global future: low-carbon economy or high-carbon economy?. World.

